# Level of knowledge on risks to HIV and AIDS among secondary school students in the Kisumu District

**DOI:** 10.1186/1742-4690-9-S1-P125

**Published:** 2012-05-25

**Authors:** JT Ongwara, Obadha Odenyo

**Affiliations:** 1 Maseno University, Private bag Siriba Maseno, Kenya; 2 Institute of Tropical Medicine, Kisumu, Kenya, Kisumu, Kenya

## Objective(s)

The objectives of the study were:

1) To assess the General knowledge and awareness among secondary school students in Winam Division, Kisumu District on: a) Modes of transmission of HIV and AIDS b) Signs and symptoms c) Prevention methods against HIV and AIDS.

## Design

The study design was descriptive and cross-sectional and was carried out in the study area between September and November 2007. Setting It covered a sample of student population of 405 drawn from 9 out of 30 schools in Winam Division. Simple random and Probability proportional to size sampling methods were used to sample the schools and the actual participants from each class.

Subjects or participants Students attending secondary schools in the study area.

## Results

About (99%) indicated that they had heard about AIDS compared to only 4 (1%) who had not. Knowledge had no statistically significant relationship with risk of HIV and AIDS. About 53.3% of the respondents reported to have had sex with males being more likely to have an early sexual debut. Sexual activity was higher among peri-urban respondents (37%) who also had more than 3 sexual partners. About 71.4% of the respondents were willing to change their behaviour to avoid contracting HIV. On bivariate analysis, exposure to risk factors was dependent on gender (p < 0.05), perceived risk and condom used were related (p < 0.05).

## Conclusion

This study concluded that despite their high knowledge and awareness on HIV and AIDS, not all students who were exposed to risk perceived themselves to be at risk.

